# Six Recommendations for Provider Behavior Change in Family Planning

**DOI:** 10.9745/GHSP-D-22-00495

**Published:** 2023-11-30

**Authors:** Heather Hancock, Olivia Carlson, Hope Hempstone, Bethany Arnold, Kamden Hoffmann, Xaher Gul, Kathryn Spielman

**Affiliations:** a Johns Hopkins Center for Communication Programs, Baltimore, MD, USA.; b Independent consultant, Bethesda, MD, USA.; c Independent contributor, Baltimore, MD, USA.; d USAID/MOMENTUM Integrated Health Resilience, IMA World Health, Washington, DC, USA.; e Pathfinder International, Watertown, MA, USA.; f Population Council, Washington, DC, USA.

## Abstract

Health care provider behavior has the power to influence family planning and reproductive health outcomes positively and negatively, underlining the importance of provider behavior change (PBC) initiatives. However, global health practitioners lack a shared understanding of PBC interventions and what influences provider behavior. Furthermore, PBC interventions in family planning and reproductive health have tended to address individual and workplace environmental factors rather than the full breadth of factors that influence provider behavior, including the broader systems and contexts where providers operate. This commentary contributes to a common understanding of PBC, including the determinants of provider behavior, and describes actions to advance PBC efforts in family planning and reproductive health. To inform these considerations, we conducted a narrative review of more than 70 articles and project materials describing interventions that aimed to change provider behaviors pertaining to family planning and reproductive health and used the review to identify the most and least common provider cadres addressed, behavioral determinants targeted, and strategies implemented. We strongly encourage global health practitioners to design future PBC interventions for a more diverse set of cadres and contexts, consider the full set of factors that influence provider behavior, pair provider- and client-side interventions, shift the narrative around PBC from “blaming” to supporting providers, move beyond training-only interventions, and improve the rigor of measurement and evidence-building efforts for PBC. These considerations can be used to advance the field of PBC in family planning and reproductive health to improve outcomes across the service delivery continuum.

## Abstract (precis)

Future provider behavior change interventions in FP/RH should engage a wider range of provider cadres and apply varied behavior change strategies, using a systems approach to address the holistic set of factors that influence provider behavior.

## Key Messages

- Global health practitioners lack a shared understanding of provider behavior change (PBC) interventions and what influences provider behavior.
- Past PBC interventions in family planning and reproductive health have focused on a small subset of provider cadres and largely addressed behavioral determinants at the individual or workplace environment levels rather than the full breadth of factors that influence provider behavior, including the broader systems and contexts where providers operate.
- We encourage practitioners to adopt a theory-driven lens and use implementation science principles to be more intentional in designing future PBC interventions for a wider diversity of cadres and contexts, consider the full set of factors that influence provider behavior, pair provider- and client-side interventions, shift the narrative around PBC from “blaming” to supporting providers, move beyond training-only interventions, and improve the rigor of PBC intervention measurement and the evidence base.

## INTRODUCTION

Health care provider behavior has the power to influence family planning and reproductive health (FP/RH) outcomes positively and negatively,^1^ underlining the importance of provider behavior change (PBC) initiatives. Since the 1970s, 2 streams of research have explored factors that influence provider behavior but have yet to converge in a way that provides meaningful and practical insights for practitioners seeking to improve provider behavior. Early literature in this period focused on identifying policies to regulate provider behaviors in the context of increased access to care.^2^ Subsequent efforts to improve quality of care described provider behavior as a mediator between interventions and improvements in quality of care. Various determinants were found to be linked to provider performance and motivation, such as policies (e.g., payments, incentives, and financing mechanisms)^3^^–^^7^; institutional mechanisms (e.g., lack of organizational support, inadequate training support, poor supervisory support, and resource shortages)^7^^–^^11^; and facility-level factors (e.g., high workload, unbalanced division of labor, and poor infrastructure).^7^^,^^12^^,^^13^

In parallel, research from the fields of health psychology and communication has examined client-provider communication and provider behaviors within the context of the client-provider relationship.^14^^–^^17^ Roter and Hall describe 4 types of interactions between providers and clients based on power and information asymmetry: paternalistic, mutuality, default, and consumerism.^16^ The literature also describes key communication functions at the provider-client interface (e.g., fostering healing relationships, information exchange, and enabling patient self-management); potential pathways to improved quality of care and health outcomes; and recommendations for developing validated measures to test these pathways of change.^16^^,^^18^ Evidence also supports the critical role of applying behavior change theories at multiple levels to account for factors such as social and gender norms, trust, community support, provider values, and provider perceptions of these factors in determining health provider motivation, communication, and performance.^9^^,^^19^^–^^23^

Although these 2 streams of research are complementary and focused on similar objectives, they have not come together in a manner that is meaningful and actionable for global health practitioners. Consequently, despite substantial investments in research on provider behavior and quality of care, global health practitioners lack a shared understanding of PBC and what influences provider behavior. Furthermore, quality-of-care interventions that aim to support positive provider behaviors are not always referred to as PBC interventions by implementers.^24^ PBC interventions in FP/RH have also tended to address individual and workplace environmental factors rather than the full breadth of factors that influence behavior, including broader systems and contexts where providers operate. This bias toward addressing the proximate factors that influence behavior rather than underlying social and structural factors is not unique to PBC interventions for FP/RH but is common across public health.^25^

Despite substantial investments in research on provider behavior and quality of care, global health practitioners lack a shared understanding of PBC and what influences provider behavior.

The purpose of this commentary is to contribute to a common understanding of PBC among global health practitioners, including a common frame of reference for identifying determinants of provider behavior, and propose several recommendations to strengthen FP/RH PBC approaches by addressing the holistic set of factors driving provider behaviors. This shared understanding of PBC concepts and priorities is particularly essential given the proliferation of PBC research and programming in recent years. To inform these considerations, we conducted a narrative review of more than 70 articles and project materials describing interventions that aimed to change FP/RH provider behavior and used the review to identify the most and least common provider cadres addressed, behavioral determinants targeted, and strategies implemented.

## WHO IS A HEALTH CARE PROVIDER?

Broadly, health care providers are individuals who provide services, products, or information to promote, protect, and improve health.^26^ Health care providers constitute a diverse group of individuals who operate in different settings with distinct roles and varied levels of training. The World Health Organization has classified these individuals into 5 categories:

1. Health professionals (e.g., medical doctors, nurses, midwives, and pharmacists)
2. Health associate professionals (e.g., community health workers [CHWs], associate or auxiliary nursing and midwifery professionals, medical assistants, and lab technicians)
3. Personal care workers in health services (e.g., health care assistants and home-based personal care workers)
4. Health management and support personnel (e.g., health service managers/administrators, social workers, counselors, and administrative and building staff)
5. Other health service providers not elsewhere classified (e.g., traditional healers and birth attendants, medical student interns, and volunteers)^27^

The term health care provider may have different connotations in different settings, particularly given country-specific legal training and certification requirements for each cadre. The insights and recommendations in this commentary are primarily informed by the available literature and documentation related to PBC programming for FP/RH health professionals, which largely focuses on formally trained and state-authorized providers who practice modern medicine. These providers include generalist or specialist medical doctors, nursing professionals, midwifery professionals, pharmacists, and CHWs working in the public or private sector, who directly provide care and/or provide management or supervision of care. Though we did not limit our review to literature describing PBC interventions that address these specific cadres, the majority of available literature pertains to these groups.

## WHAT IS PROVIDER BEHAVIOR?

Provider behavior is defined as what providers do and do not do in their professional capacity. Specific provider behaviors depend on the provider cadre, their scope of practice, the health topic/technical area of focus, and other context-specific conditions, including the time frame during which that behavior must be performed.^28^ Some behaviors are enacted during the client-provider encounter and directly influence quality of care and client health outcomes. Other, more “upstream” behaviors precede the client-provider interaction but have a bearing on provider motivation, capacity, competence, and performance.^29^^–^^31^ Studies also suggest the behavior of supervisors and managers may also impact that of providers charged with direct client care, ultimately influencing client experience (Table 1).^32^^,^^33^

**TABLE 1.** Examples of Recommended Provider and Supervisor Behavior Categories^a^

Provider Behaviors		Supervisor Behaviors
Before the Client-Provider Encounter	During the Client-Provider Encounter	
Self-reflecting Communicating with colleagues Managing facilities, stock, and supply Pursuing professional development opportunities	Conducting systematic assessments to support sound clinical decision-making Adhering to clinical guidelines Counseling or client education Keeping records Managing work- and client-flow Collaborating with colleagues Making referrals Encouraging client communication and question asking	Providing actionable feedback Establishing and enacting accountability mechanisms Facilitating continuous learning and advancement opportunities

a These are summary behavior categories that need contextual specification for effective provider behavior change programming. Practitioners should consider provider cadre, their scope of practice, the health topic/technical area of focus, and other context-specific conditions when identifying the specific behavior or set of behaviors they aim to change.

How these behaviors are enacted is as important as whether they occur. Person-centered care requires providers to interact with clients in a way that is respectful, individualized, and empowering. Although FP/RH service provision standards typically reflect these principles,^34^ they are not consistently applied in practice, given norms in the facility and the community, time constraints, and other factors.

Provider behavior is influenced directly and indirectly by an array of individual, interpersonal, social, and structural factors and the complex interactions between them.^35^^–^^36^ Understanding the factors that drive provider behavior—and the relationships between those factors—is essential to designing and implementing effective interventions. The Provider Behavior Ecosystem Map identifies 7 “influence factors” or “overarching groupings of key actors, entities, and other elements that interact with providers and influence provider behavior”: the individual, personal relationships, the client, community context and social norms, workplace environment, health system governance, and country and geopolitical context (Table 2).^12^

**TABLE 2.** Provider Behavior Influence Factors^a^

Influence Factor	Description
Individual	Provider characteristics, history, experience, and professional purpose (e.g., provider's attitudes, knowledge, personality type, gender competency, and goals).
Personal relationships	A provider's personal relationships with partners, family, friends, mentors, colleagues, instructors, and community leaders (e.g., the gender norms and attitudes that affect relationships between providers and their partners, as well as the attitudes and beliefs their friends and family hold related to sexuality and contraception).
Client	The client's personal characteristics, history, and health situation (e.g., identity, health literacy, expectations for care, agency, emotional activators, and perceptions).
Community context and social norms	People and community structures, community and social characteristics (including social norms), and the health care delivery context in the community (e.g., community organization, accountability measures, gender and social norms, social stigma, discrimination, health mis/disinformation, and community-facility dynamics).
Workplace environment	People working at a facility and their interactions, the culture of the facility, its infrastructure, and workplace governance (e.g., hierarchy and power dynamics, staffing levels and workload, perceived support, leadership and management, physical environment, and facility type).
Health system governance	Quality assurance, health care delivery process and practice, and leadership (e.g., provider support structures, resource management, health care costs, policies, and health system culture).
Country and geopolitical context	Broad national conditions in the country, health care delivery enablers, and rules and assurances (e.g., enforcement and compliance, political context and priorities, donor ideologies and incentives, and the social and economic context).

a Source: Breakthrough ACTION. Provider Behavior Ecosystem Map.^35^

Understanding the factors that drive provider behavior—and the relationships between those factors—is essential to designing and implementing effective interventions.

Factors influencing provider behavior are complex and interrelated. For example, providers' perceptions of the level and type of support they receive from supervisors may influence providers' self-efficacy, job satisfaction, and motivation to provide quality services. The extent to which FP/RH services are prioritized and funded within the health system may affect providers' compensation, staffing levels, workload, perception of support (or lack thereof), morale, and motivation.^12^

The factors that most strongly influence a given behavior are specific to the provider profile (i.e., cadre, experience, age, gender, and other characteristics) and the social and structural context where providers practice.^37^ Key differences between paid and unpaid health workers, for example, may affect behavior, including motivations and levels of training and education.^38^ There are also distinctions between community- and facility-based providers who operate in physical and management environments with varying structural, organizational, and workplace environment considerations. Factors such as time commitment and incentives/remuneration, the extent to which CHW programs are integrated into health systems, and the level and quality of collaboration between CHWs and other actors in the health system—including linkages to a supportive and functioning referral facility—all influence CHW effectiveness^39^ but may be less relevant for their facility-based counterparts.

## WHAT ARE PBC INTERVENTIONS?

PBC interventions are defined as those aiming to support providers to improve client health outcomes by addressing factors that shape provider behavior (Table 2). These interventions may engage directly with providers to address individual and interpersonal influence factors and/or indirectly to address client, community, workplace, health systems, and country or geopolitical influence factors that impede or facilitate positive provider behaviors.

PBC interventions apply learnings from the service delivery and social and behavior change (SBC) fields. Service delivery approaches improve access to quality health services by strategically increasing health system inputs (e.g., health workers, finances, policy, and supplies and procurement),^40^ and SBC approaches use a deep understanding of human and societal behavior to promote positive behaviors by addressing the individual, interpersonal, social, and structural factors that underpin them.^41^ Expertise and partnership from both disciplines are required to design and implement effective PBC strategies that improve access to quality health services, client health outcomes, and a client's experience of care.

While significant progress has been made in the study and application of PBC approaches in the past several decades, practitioners and researchers have often failed to clearly define the term PBC or have not used it to describe interventions aiming to improve provider behavior. PBC interventions have often been corrective and focused on changing undesirable provider behaviors^42^ rather than promoting positive ones. Many PBC interventions have been narrow in their application, focusing on individual- or workplace-level behavioral drivers (Table 2) rather than combining those approaches with those that would influence broader health systems and structural determinants of provider behaviors.

## SIX RECOMMENDATIONS FOR FUTURE FP/RH PBC PROGRAMMING

Building from this understanding of providers, provider behavior, and past FP/RH PBC efforts, we propose the following recommendations to advance FP/RH PBC programming.

### 1. Address Diversity of Providers and Their Environments

Practitioners should acknowledge and address the diversity of providers and the environments and systems in which they operate. Many FP/RH PBC interventions target facility-based doctors, nurses, and midwives. Although these providers are important, they are but a few of the many cadres that influence client outcomes. Global health practitioners must address a broader set of providers, including auxiliary and informal cadres and those not directly associated with health facilities, and seek to better understand their unique needs and perspectives (Box 1).^43^ Building on that understanding, practitioners need to identify and prioritize the specific provider behaviors they are seeking to change, ensuring the behaviors are cadre and context appropriate. Equally important is understanding and tackling power dynamics within a facility, which can hamper PBC. Power dynamics reflect established hierarchies within medicine but may also be influenced by cadre, gender, age, experience, race, ethnicity, and other factors.^44^^,^^45^ For providers of all cadres, clearly defining professional roles and responsibilities supports efficiency and collaboration; this foundational activity is particularly critical in the context of task-shifting.^46^

Global health practitioners must address a broader set of providers, including auxiliary and informal cadres and those not directly associated with health facilities, and seek to better understand their unique needs and perspectives.

BOX 1 Segmenting Provider Audiences to Better Address Diverse Needs and Perspectives

“Audience segmentation is a key activity within an audience analysis. It is the process of dividing a large audience into smaller groups of people—or segments—who have similar needs, values, or characteristics. Segmentation recognizes that different groups will respond differently to [SBC] messages and interventions.”^43^

For example, provider cadre (doctor, nurse, and midwife) may be linked to socioeconomic status, social class, or other sociodemographic characteristics that have a profound impact on behavior. Audience segmentation guidance can be found on the Compass website (https://www.thecompassforsbc.org/).

Research suggests that interventions that bring together all facility staff or multiple cadres of providers and facility support personnel may be especially effective in improving inter-cadre trust and teamwork, increasing provider satisfaction and motivation, and, thus, changing behavior.^47^^,^^48^ One reason for the success of such interventions may be that they increase “connectedness” within and across cadres at multiple levels of the health system. Interventions engaging providers and supervisors (recognizing that a single professional may play both roles) also offer promise, as they leverage the ability of supervisors to model positive norms and behaviors, provide technical guidance and mentorship, and monitor the practice of desired behaviors (Box 2).^48^

BOX 2 Example of Efforts to Engage Health Facility Staff at Multiple Levels

The Ghana Vasectomy Initiative engaged health facility staff at all levels to improve knowledge and acceptance of no-scalpel vasectomy (NSV). During 2003–2004 and 2007–2008, the Ghana Health Service partnered with EngenderHealth to improve knowledge, acceptance, and use of NSV by training health staff at all levels on providing “male-friendly” services, training physicians in NSV provision, and implementing client-centered health promotion activities. The whole-site trainings engaged both medical and nonmedical staff—“including physicians, nurses, midwives, community health workers, health educators, receptionists, cleaning staff, and guards”—to create a welcoming environment for male clients and improve the quality of NSV counseling.^48^

After the whole-site training, facility staff held more positive attitudes “toward providing reproductive health services for men,” had improved knowledge of male anatomy, held fewer misconceptions about vasectomy, and felt more comfortable “talking with men about family planning.”^48^ The whole-site trainings, coupled with client-facing health promotion activities, was associated with a three-fold increase in uptake of NSV services from 2003 to 2004 (26 procedures in 2003 to 83 in 2004) and 2007 to 2008 (18 procedures in 2007 to 53 procedures in 2008).^48^

### 2. Expand Programming to Address Diverse Drivers of Provider Behavior

Global health practitioners must adopt a systems approach, expanding programming to comprehensively address diverse drivers of provider behavior. This expansion is crucial in areas with historic gaps at the interpersonal, social, and structural levels. FP/RH PBC interventions often focus on determinants of provider behavior at the individual or workplace environment level; fewer have addressed the sociocultural, structural, or geopolitical factors that influence behavior. Evidence demonstrates that PBC strategies are more effective when paired with interventions that address behavioral determinants at different levels, such as supervision with mentoring and community engagement, or infrastructural improvements with management changes and training.^1^^,^^49^^,^^50^ At the structural level, studies emphasize the need to ensure more supportive policies and guidelines for both providers and clients, particularly those influencing staffing, provider roles, supervision, measurement and accountability, payment mechanisms, and training.^51^^,^^52^ Similarly, closing structural gaps to ensure reliable supplies of contraceptive commodities and equipment, adequate staffing levels, conducive physical environments, and access to systems and process management tools can support providers to operate optimally. Although it can be inferred that past “supply-side” interventions to improve quality of care—including youth-sensitive policy reforms and curricular revisions—aimed to change provider behavior, many did not explicitly identify PBC as an indirect goal. Practitioners and researchers need to make greater efforts to identify and measure the relationships between these structural-level interventions and specific provider behaviors.

At the social level, initiatives must acknowledge the strong influence that sociocultural norms, community-level factors, and gender norms—with associated sanctions and stigma—have on providers, clients, and the health system.^35^^,^^53^^,^^54^ For example, providers are less likely to perceive clients with lower socioeconomic status as intelligent and capable of following medical advice,^55^ and these biases influence clinical decision-making and patient care.^56^^–^^60^ To be effective, PBC interventions must address these factors directly alongside other drivers of provider behavior.^61^ Literature highlights several promising strategies, including social accountability mechanisms, social norms change, community dialogues, and organized diffusion.^62^^,^^63^ Finally, given that knowledge and skills are inadequate predictors of provider behavior,^64^^,^^65^ individual-level initiatives should focus on provider factors, including motivation, attitudes, biases, perceived norms, and identity, linking the influence of initiatives at structural and social levels to these individual-level drivers of provider behavior.^66^

The application of behavior change theory and established frameworks can be particularly helpful for service delivery implementers in realizing this recommendation. Using a theory-driven approach can help implementers map and address key determinants of provider behavior in a clearly defined pathway of change.

### 3. Combine Provider- and Client-Side Interventions

Given the dyadic nature of provider-client interaction, global health practitioners implementing PBC activities should pair provider-focused interventions with client-focused interventions. Despite evidence that client factors influence provider behavior, clients are rarely a focus of PBC activities.^67^^,^^68^ Clients' previous health care experiences, perceptions of providers, attitudes, health literacy, and self-efficacy directly impact their expectations for care and how they approach provider interactions.^35^ Readiness for an interaction can influence what and how clients share information with providers.^69^ Data on client-side activities are limited, but studies suggest that interventions that prime clients to ask questions and share preferences or past histories may support positive provider behavior and improve the overall interaction.^70^^,^^71^ Peer support groups have shown promise for trusted information sharing and client empowerment in preparation for interacting with a provider.^72^ PBC interventions may broadly benefit from activities seeking to shift client attitudes, bolster self-efficacy, and foster empathy between clients and providers.^48^ Client features, including demographics (particularly age) and ability to pay, can activate provider attitudes and biases and shape how a provider treats clients.^73^^,^^74^ PBC interventions that promote client-centered care and build provider skills to offer tailored, respectful, and empowering care can address some of those barriers.^75^ Similarly, efforts to help providers recognize and name biases, such as values clarification exercises,^42^^,^^76^ may build self-awareness and prompt action to improve behavior. Beyond the individual level, PBC interventions that increase providers' empathy toward clients and address normative barriers and stigma at the social level can also lead to positive provider behaviors.^77^^–^^80^ In sum, PBC interventions must address both sides of the equation to effectively support positive provider behavior rather than remaining siloed (Box 3).

PBC interventions must address both sides of the equation to effectively support positive provider behavior rather than remaining siloed.

BOX 3 Example of a Provider Behavior Change Intervention That Addressed Provider- and Client-Side Factors

The Quality Improvement Initiative in Indonesia implemented provider- and client-facing activities to improve the provider-client interaction.

The provider-facing arm included training for providers on client-centered counseling, self-assessments, and peer-review meetings. Pairing the training with self-assessments was associated with increases in “providers' facilitative communication, clients' active communication, and clients' ratings of self-expression and satisfaction.”^69^

The initiative also implemented a complementary client-facing education and mass media intervention that saw improvements in “the number of clients who prepared questions to ask the service provider prior to a family planning visit” and overall client participation in family planning counseling sessions.^71^ The Quality Improvement Initiative highlights the need for both client- and provider-facing provider behavior change activities to positively influence the provider-client interaction.

### 4. Shift from Blaming to Supportive Problem-Solving

Researchers and practitioners must shift the PBC narrative from blame to accountability by empowering providers, promoting provider autonomy, and pursuing large-scale policy and procedural changes. While the drivers of provider behavior vary widely, those engaging in PBC efforts must begin with the assumption that, with few exceptions, providers wish to support their clients in achieving good health. At the most basic level, this may require changing the language used to describe PBC to emphasize motivation and performance rather than “correction.” This positive framing is equally important in designing interventions. Research shows that providers often experience management and supervision as punitive; this culture of blame is a powerful demotivating force.^81^^,^^82^ Efforts to improve quality of care, including those that rely upon training and supportive supervision, should emphasize collective problem-solving and accountability with providers and communities^83^ rather than deficiencies. Empowering providers to identify and overcome barriers is critical to individual performance, team effectiveness, and quality of care. This will be a culture shift since promoting real provider empowerment may require advocacy for changes to policies and procedures that influence access, opportunities, and the larger culture.

Efforts to improve quality of care should emphasize collective problem-solving and accountability with providers rather than deficiencies.

### 5. Move Beyond Training-Only Approaches

Practitioners must acknowledge that training is insufficient for sustained PBC.^1^ PBC interventions can extend learning and application if training is complemented by efforts to improve supply and management of commodities; changes to health care policy, staffing, workload, or workflow; or demand creation, client empowerment, and community support for quality services.^84^ In many settings, low-cost, close-to-practice training that allows for peer-to-peer learning and reinforcement through supervision and mentoring may be more effective than offsite courses.^85^ Reinforcing behaviors through rewards and incentives, including recognition programs, can increase provider motivation.^86^^,^^87^ Training should couple instructional learning with more interactive approaches and must include opportunities for learners to routinely practice newly acquired skills.^85^ The latter may be achieved through exercises such as role-plays or simulations during the training, as well as efforts to increase the use of services and ensure regular client flow afterward. Finally, provider training must address not only clinical knowledge and skills but essential “soft” skills, including interpersonal communication, active listening, strategies for engaging effectively with different client groups and addressing difficult situations, time management, and supervision.

### 6. Improve the Rigor of PBC Measurement and Expand the Evidence Base

Global health practitioners should improve the rigor of PBC measurement and expand the evidence base in FP/RH by prioritizing and budgeting for the monitoring, evaluation, and documentation of PBC activities. This includes understanding which PBC strategies work, in what sequence, and in which contexts. Specifically, more insight is needed into what client-side strategies might prompt improvements in provider behavior and effective strategies for including communities as partners in PBC. Work should build on current efforts to understand the outcomes and impact of social accountability mechanisms such as report cards, community scorecards, quality improvement teams (inclusive of providers and community members), and community health committees. To improve the rigor of measurement, implementers should articulate a change pathway in advance, with theory-driven outcomes at different levels, to describe causal relationships between different behavioral drivers and formulate learning questions; develop, test, and validate theory-driven scales and other standardized approaches to measure changes in behavioral drivers for providers across influence factors and diverse provider cadres; and use these validated measures to confirm these relationships and evaluate outcomes (Box 4).

BOX 4 Recommendations for Improving the Rigor of Provider Behavior Change Interventions

1. Develop a theory of change to describe a change pathway.
2. Describe relationships between the influence factors and outcomes in the change pathway. Consider the following wide range of provider behavior change measures, listed in order of hierarchy in the change pathway:
  - Specific provider behavior to change
  - Provider attributes, including sociocultural characteristics and psychosocial drivers of behaviors (e.g., intention, attitudes, perceived norms, personal agency, knowledge, and skills, including communication skills)
  - Client-level factors and the quality of the provider-client interaction
  - Facility infrastructure and the service delivery environment
  - Institutional mechanisms, including the availability of continued learning opportunities and supportive supervision; accountability mechanisms; incentive mechanisms; and information management systems and use
  - Existing mandates, policies, and regulations related to the behavior
3. Develop, test, and validate theory-driven scales and other standardized approaches to measure provider behavior change outcomes.
4. Apply validated measures to monitor change and performance, evaluate outcomes at different levels, and confirm causal relationships along the change pathway.

Breakthrough ACTION is compiling a list of PBC indicators for measurement at each level of the Provider Behavior Ecosystem^35^ and synthesizing guidance for measuring provider behavior. Once available, practitioners should use this guidance, further validate the available indicators, and document their use to build the evidence base around PBC measurement. Three examples of rigorous measurement tools/indices include the “Provider Authoritarian Attitude Scale,”^88^ “Perceived Person-Centeredness of FP Care Scales,”^89^ and the “Twelve Recommended SBC Indicators for Family Planning.”^90^ Further, expanding documentation of learning and experiences around PBC for different cadres of providers is needed, including the use of adaptive management techniques. For example, Scott et al. note that most evaluations of CHW programming include very little programmatic detail (e.g., recruitment strategy, eligibility criteria, training content, supervision, incentives, and reporting).^39^ Absent this type of information, expanding the evidence base for PBC—or even identifying interventions that include PBC components—will be difficult.

## CHARTING THE WAY FORWARD

This commentary provides recommendations for PBC programming based on learnings from the current literature. However, applying a holistic systems lens to FP/RH PBC efforts is relatively nascent, and much remains unknown. The following areas require further exploration and collaboration between global health practitioners.

- **Defining Provider Behaviors and Deepening Appreciation of Its Influences.** Much of the current literature on FP/RH provider behavior considers processes comprising either multiple behaviors (such as “high-quality counseling”) or behaviors specific to a particular tool. Defining priority provider behaviors distinguishes PBC initiatives from older approaches to performance improvement. Defining these specific behaviors in terms of action, context, and time frame^28^ allows us to identify key audiences and behavioral determinants and accurately measure intervention effectiveness. More work must be done to define specific behavior profiles, guide the prioritization of behaviors, and determine what influences those provider behaviors in various contexts. We must also explicitly link efforts to improve provider behavior with client health outcomes.
- **Understanding the Needs of and Synthesizing Recommendations for a Broader Group of Providers.** Due to the current focus of the literature, this commentary did not formulate recommendations for private sector facilities, pharmacists, informal providers, facility support personnel, or traditional healers. There are also key differences between paid and unpaid providers, CHWs and facility-based providers, and different cadres of facility-based providers that need to be further investigated.
- **Understanding What PBC Strategies Work in Which Contexts and for Which Cadres**. In particular, more evidence is needed to support the emergent use of digital solutions,^91^^,^^92^ interventions that address both clients and providers, social accountability approaches,^93^ and connections between systems-level strategies and provider behavior. To advance the field, we also need to narrow the gap between what is currently measured and what theory suggests should be measured.
- **Aligning on What Constitutes PBC.** In FP/RH, PBC has often been equated with addressing provider bias or building interpersonal communication skills, which is a narrow and oversimplified conception. Those working with providers—including those in other health areas—must come together to deliberately define PBC and discuss what is needed to support providers in advancing the practice of respectful and person-centered care across the life course.
- **Understanding Gender's Role in PBC Efforts Between Providers and Clients and Among Providers.** Some articles examined the acceptability of female CHWs^94^^,^^95^ but did not address the role of gender norms and dynamics in PBC, including gender dynamics in a team of providers. They also did not explore how gender influences the health system and provider practices, including work that supports providers to interact with women, men, girls, boys, and couples together. It is critical to continue exploring how gender influences vary by context and to reflect on what that means for PBC. Practitioners need to apply an intersectional lens to PBC efforts and deliberately consider gender influences during design, implementation, and evaluation.

More work must be done to define specific behavior profiles, guide the prioritization of behaviors, and determine what influences those provider behaviors in various contexts.

## CONCLUSION

This commentary contributes to a common understanding of PBC, including the determinants of provider behavior, and makes several recommendations to advance FP/RH PBC efforts. We encourage practitioners to adopt a theory-driven lens and use implementation science principles to be more intentional in the design of future PBC interventions for a wider diversity of cadres and contexts, consider the full set of factors that influence provider behavior, pair provider- and client-side interventions, shift the narrative around PBC from “blaming” to supporting providers, move beyond training-only interventions, and improve the rigor of PBC intervention measurement and the evidence base.

FP/RH provider behavior heavily influences the quality of health services and a client's experience of care. SBC and service delivery practitioners have distinct but complementary roles and expertise required to design and implement effective PBC interventions, and health care providers themselves should be engaged as skilled partners who share the common goal of improving the health and well-being of their clients. Beyond individual FP/RH PBC interventions, large-scale, sustained improvements in provider behavior will require changes in several areas, including pre-service training, clinical practice guidelines, and health management information systems, each of which requires the engagement of academic institutions, professional groups, normative bodies, and government structures at the national level. Cultivating partnerships between government decision-makers, activists, communities, researchers, the media, medical professionals, and other key stakeholders can draw attention to the relationship between provider behavior, client satisfaction and utilization of services, and health outcomes, as well as garner support for localized PBC interventions.

## Notes

**Peer Reviewed**

**First published online:** August 23, 2023.
